# A Textbook of General Therapeutics

**Published:** 1889-11-30

**Authors:** 


### PRACTITIONER'S BOOKSHELF.

### A TEXTBOOK OF GENERAL THERAPEUTICS.

This work* is on the basis of the author's lectures delivered at Guy's Hospital. The first chapter is devoted to medical climatology, and treats of the points to be considered in selecting a climate for an invalid, which are purity of atmosphere, temperature, humidity, sunlight, rarefaction, ozone, wind, electricity, soil, and vegetation. This chapter affords exactly the information required by students, or by practitioners desirous of refreshing their memories on the points treated of. A similar observation applies to chapter ii. "On the Classification of Climates." Chapter iii. is headed "Treatment of Disease by Climate." Here we have discussed the effect of high altitudes on phthisical patients. The distinguishing features of the climates of high altitudes are purity of air, comparative high temperature in winter as a result of stillness of air, little wind, dryness of atmosphere, increased light from absence of clouds and moisture, increased quantity of ozone, dry soil, altered electrical conditions, dimunition of barometric pressure. The effect of all these conditions are considered, and "each of them is probably beneficial for phthisis." But it is advisable that only suitable cases should be sent to high altitudes, and a list is given of those cases which should not go to mountains. Chapter iv. is on "Treatment by Compressed Air," according to Oertel's plan. The theory is, that inhaling compressed air dilates the thorax and the lungs to the utmost. Collapsed alveoli are distended, and air passes easily into tubes narrowed by mucus or swelling. Benefit to different maladies has been reported from this treatment, but as it "occasionally leads to catarrh of the bronchial mucous membrane," much caution is required in its application. The next chapter, v., is on "Oertel's Method of Treating Chronic Cardiac Disease." This consists of a combined system of diet and exercise. But it is more applicable to cases of fatty heart than any other cardiac malady. Patients are to walk up hills, drink little fluid, and take a diet consisting chiefly of proteids. We have not space to enter into pros and cons. of this subject, which the enquirer will find fully considered in Dr. Hale White's book. Chapter vi. is headed "Diet," and will well repay perusal. Holding in recollection a communication from Dr. G. Herschell, wherein he questions the teachings of textbooks that human beings cannot live on a meat diet, we turn with some curiosity to see what Dr. Hale White says on this matter. At page 68 we find, "Flesh which only contains albumen, water, and salts, is the single food which can support life any length of time . . . but it is not a suitable diet, because of the excess of proteid, and because of the indigestion it induces." Chapter vii. is devoted to "Diet in Different Diseases." Chapter viii. is on "The Drinking of Water," from which we glean that while drinking either hot or cold water suits some persons, it does not agree with others, and therefore generalisation is impossible. One point, however, is clear—viz., that if a great quantity of water is drunk more than the usual amount of urea is excited, so that we readily arrive at the conditions in which more water drinking is desirable. In the same chapter mineral waters are discussed. Chapter ix. relates to baths and other external applications of water ; and chapter x. to cold water as an antipyretic. The author seems to favour the treatment of febrile conditions by cold baths. But we have known dangerous collapse result. Moreover, there is the query, never satisfactorily answered, whether good results from temporary reduction of temperature in fever. A certain amount of destruction of tissue must occur during an attack of fever, and this metabolism is delayed, not prevented, by cold. On the other hand it is asserted that excess of heat alone will destroy life. The conclusion is, that the cold bath, if used at all, must be given with great caution. Chapter xi. relates to artificial baths, as the hot air and the Turkish bath. Chapter xii. is on "Lavage, or Washing out the Stomach." A plate is introduced, showing how this is accomplished by a syphon arrangement. The next chapter, xiii., is on "Massage," of which a great deal too much has been made, being as it is, simply an extension of the shampooing practised for ages by the natives of the East. Chapter xiv. is on the "Weir-Mitchell Method," which consists of isolation of the patient, rest in bed, massage, electricity, and over-feeding. The class of patients best suited to this method are neurotic hysterical women, in whom there is much loss of flesh. Severe organic diseases contraindicate it. "Venesection" is the title of chapter xv. In common with many other practitioners, the author seems to think that blood-letting is too much neglected as a curative agent in the present day. Then we have chapter xvi. on "Electricity," and chapter xvii. on "Electrodiagnosis," copiously illustrated by plates. Chapter xix. is headed "Hypnotism," and the author does not forget to mention "the dangers of the hypnotic state." The concluding sections are on "Metallo-therapy," on "Treatment of Diseases of the Spinal Cord by Suspension," and on "The General Treatment of Acute Diseases, Convalescence, and Insomnia." The value of the book is enhanced by a copious index. We may honestly recommend Dr. Hale White's textbook of general therapeutics, as containing a vast fund of information not, we believe, ever before brought together in any one volume. The author states that to prepare it a very great many books, pamphlets, and articles have been read, and it may be mentioned that a bibliography is attached to each subject. Dr. Hale White acknowledges the assistance received from Dr. Ryle, Dr. Pitt, Dr. A. T. Myers, and from several authors who have allowed him to copy woodcuts.

* "A Textbook of General Therapeutics." By W. Hale White, M.D., F.R.C.P., Senior Assistant Physician and Lecturer on Materia Medica and Therapeutics at Guy's Hospital. With illustrations. Macmillan and Co., London and New York. 1889.

